# Multicentre studies of insecticide-treated durable wall lining in Africa and South-East Asia: entomological efficacy and household acceptability during one year of field use

**DOI:** 10.1186/1475-2875-11-358

**Published:** 2012-10-29

**Authors:** Louisa A Messenger, Abrahan Matias, Antonio Nkulu Manana, Joseph B Stiles-Ocran, Steve Knowles, Daniel A Boakye, Mamadou B Coulibaly, Marie-Louise Larsen, Amadou S Traoré, Bréhima Diallo, Mamadou Konaté, Amadou Guindo, Sékou F Traoré, Chris EG Mulder, Hoan Le, Immo Kleinschmidt, Mark Rowland

**Affiliations:** 1 Faculty of Infectious Tropical Diseases, London School of Hygiene and Tropical Medicine, London, UK; 2 Medical Care Development International (MCDI), Malabo, Equatorial Guinea; 3 Entomology Research Unit, Malaria Control Centre, AngloGold Ashanti Ltd., Obuasi, Ghana; 4 Noguchi Memorial Institute for Medical Research, UG, Accra, Ghana; 5 Vector Genomics and Proteomics, Malaria Research and Training Center (MRTC), University of Sciences, Techniques and Technologies, Bamako, Mali; 6 Technical Institute of Denmark (DTU), Lyngby, Denmark; 7 Agricultural Research Station (Pty) Ltd., Friedenhiem JT 282, Nelspruit, South Africa; 8 Vestergaard Frandsen Laboratories, 253/9 Minh Khai, Hai Ba Trung district, Hanoi, Vietnam

**Keywords:** Durable wall lining, ZeroVector®, IRS, Malaria control, Acceptability, Efficacy, Durability

## Abstract

**Background:**

Indoor residual spraying (IRS) is a primary method of malaria vector control, but its potential impact is constrained by several inherent limitations: spraying must be repeated when insecticide residues decay, householders can tire of the annual imposition and campaign costs are recurrent. Durable lining (DL) can be considered an advanced form of long-lasting IRS where insecticide is gradually released from an aesthetically attractive wall lining material to provide vector control for several years. A multicentre trial was carried out in Equatorial Guinea, Ghana, Mali, South Africa and Vietnam to assess the feasibility, durability, bioefficacy and household acceptability of DL, compared to conventional IRS or insecticide-treated curtains (LLITCs), in a variety of operational settings.

**Methods:**

This study was conducted in 220 households in traditional rural villages over 12-15 months. In all sites, rolls of DL were cut to fit house dimensions and fixed to interior wall surfaces (usually with nails and caps) by trained teams. Acceptability was assessed using a standardized questionnaire covering such topics as installation, exposure reactions, entomology, indoor environment, aesthetics and durability. Bioefficacy of interventions was evaluated using WHO cone bioassay tests at regular intervals throughout the year.

**Results:**

The deltamethrin DL demonstrated little to no decline in bioefficacy over 12-15 months, supported by minimal loss of insecticide content. By contrast, IRS displayed a significant decrease in bioactivity by 6 months and full loss after 12 months. The majority of participants in DL households perceived reductions in mosquito density (93%) and biting (82%), but no changes in indoor temperature (83%). Among those households that wanted to retain the DL, 73% cited protective reasons, 20% expressed a desire to keep theirs for decoration and 7% valued both qualities equally. In Equatorial Guinea, when offered a choice of vector control product at the end of the trial (DL, IRS or LLITCs), DL consistently emerged as the most popular intervention regardless of the earlier household allocation.

**Conclusions:**

Just as long-lasting insecticidal nets overcame several of the technical and logistical constraints associated with conventionally treated nets and then went to scale, this study demonstrates the potential of DL to sustain user compliance and overcome the operational challenges associated with IRS.

## Background

The renewed effort to control malaria is founded on the latest generation of preventative strategies and treatment options. The World Health Organization (WHO) Roll Back Malaria partnership currently recommends the use of four key interventions: long-lasting insecticidal nets (LLINs), artemisinin-based combination therapy (ACT), indoor residual spraying of insecticide (IRS) and intermittent preventive treatment in pregnancy (IPTp) [1]. Vector control using LLINs and IRS has already achieved considerable reductions in malaria morbidity and mortality [2,3]. However, the continued success of both interventions is contingent on surmounting a number of operational challenges. The impact of LLINs is heavily reliant on significant behavioural changes amongst recipients to ensure the most vulnerable individuals sleep under the nets at night [4]. In addition, community-wide coverage of LLINs is essential to suppress disease transmission and reduce exposure to unprotected persons [5,6]. IRS has demonstrated comparable levels of protection against malaria with less dependence placed on behavioural compliance [7,8]. However, overcoming user fatigue during repeated rounds of spraying in rural areas afflicted by seasonal malaria remains a challenge [9].

Recently, insecticide-treated plastic sheeting (ITPS) has emerged as a promising alternative vector control product to provide protection against endophilic vectors and nuisance insects. ITPS was originally developed to control malaria in complex emergencies by exploiting utilitarian shelter material as an insecticide delivery mechanism [10,11]. Successful control of outdoor vectors using deltamethrin-treated tarpaulins in refugee camps [12,13], coupled with the widely recognized logistical constraints associated with spraying campaigns, has initiated the use of ITPS or durable lining (DL) indoors, fixed to walls and/or ceilings, as a long-lasting alternative to IRS. Previously, ITPS applied as an interior wall lining has demonstrated an impact on disease transmission, reducing malaria incidence by over 70% in India [14].

DL is currently manufactured commercially (ZeroVector®, Vestergaard Frandsen, Switzerland) as a thin sheet of woven high-density polyethylene (HDPE) shade cloth with insecticide incorporated during production; it is designed to cover interior walls and remain efficacious for three to four years. ZeroVector® DL is based on long-lasting net technology where deltamethrin is incorporated into the polymer before yarn extrusion, allowing it to migrate to the surface in a controlled fashion and ensuring uniform coverage, regardless of surface texture or wall shape. Following initial house installation, DL requires few behavioural adjustments and adds aesthetic value to the rural home interior, thereby encouraging sustained user cooperation.

This multicentre field trial was conducted in traditional rural villages in Equatorial Guinea, Ghana, Mali, South Africa and Vietnam and is the first study to evaluate the feasibility, durability, bioefficacy and household acceptability of ZeroVector® DL compared to conventional IRS, in a variety of operational and cultural settings.

## Methods

### Study sites and vector control installation

**Figure 1:**
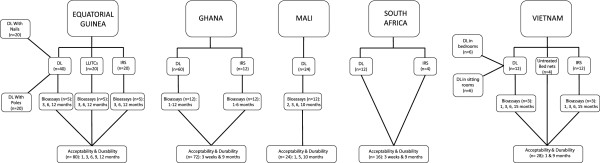
Study site profiles (n= number of households).

This multicentre study was conducted in traditional rural villages in Equatorial Guinea, Ghana, Mali, South Africa and Vietnam. The duration of this trial was for one year from August 2008 in all African sites and for fifteen months from March 2009 in Vietnam. The ZeroVector® DL installed at each study site was a thin sheet of woven HDPE shade cloth with deltamethrin (4.4 g/kg ± 15% *a.i.*) incorporated during production (supplied by Vestergaard Frandsen, Switzerland). Each roll of DL was approximately 2.3 × 100 m and was cut by a three-person installation team to fit the specific house or room dimensions. Once all pieces of furniture were moved into the centre of the room and wall items were removed, the installation team worked around the room fixing the DL to all interior wall surfaces with nails and caps at regular 60-70 cm intervals. The material was cut to fit around doors and windows and attached to the base of the roof, covering the eaves. Additionally, in Equatorial Guinea, installation of DL was trialed in a group of households using bamboo poles sourced locally. The PermaNet® long-lasting insecticide-treated curtains (LLITCs) evaluated in Equatorial Guinea measured 1.5 × 1.5 m and were made from 100% polyester netting coated with deltamethrin (1.8 g/kg ± 10% *a.i.*; also supplied by Vestergaard Frandsen, Switzerland). In study areas where IRS was evaluated as a control intervention, there was no concurrent national spray campaign occurring at the time of this trial; the only IRS was applied by project staff in selected houses. For clarity, study design is described for each site individually (Figure 1).

### Equatorial Guinea

Equatorial Guinea, situated in the Gulf of Guinea in West Central Africa, consists of a continental area (Río Muni) and an insular region (Bioko as the principal island). This study was conducted in Nsogo-Angok and Ankua (1°20’N, 10°7’W), which are rural villages located in Río Muni. The climate is tropical, characterized by two dry seasons from December to March and June to September and two rainy seasons from April to May and October to November. In Río Muni, malaria transmission by *Anopheles gambiae s.s*. is meso/hyperendemic, with prevalence rates of over 59% for children under five years old [15-17]. Following the discovery of offshore oil reserves in the mid 1990s, rapid urbanization and economic growth led to investments in public health infrastructure, including the establishment of the Bioko Island Malaria Control Project (BIMCP). Considering the success achieved by the BIMCP, malaria control was extended to Río Muni under the Equatorial Guinea Malaria Control Initiative (EGMCI) by a staged distribution of LLINs and IRS over a five year period from 2006 to 2011.

**Figure 2:**
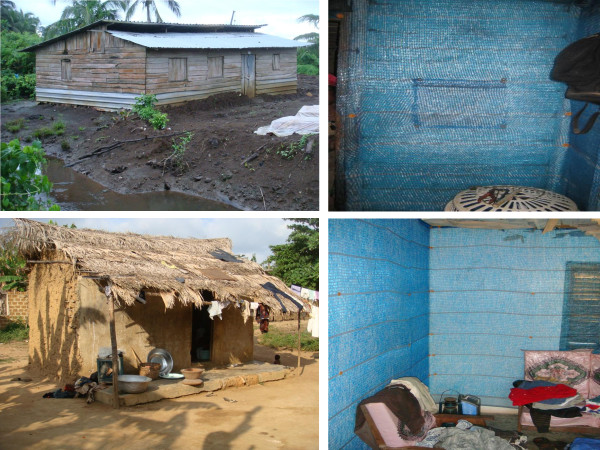
Durable linings (right) installed in traditional houses in rural Equatorial Guinea (top left) and Ghana (bottom left).

In Equatorial Guinea three vector control products were evaluated among 80 households. In 20 houses blue coloured DLs were installed using nails and caps as fixings and in 20 houses blue DLs were fitted using bamboo rods (Figure 2). PermaNet® LLITCs were distributed to an additional 20 houses. Lastly, all interior walls of another 20 houses were sprayed once at the beginning of the trial with Fendona® 5WP (5% w/w alphacypermethrin) (BASF) at an application dose of 35 mg/m^2^ using a standard 10 L Hudson X-pert pump. All spraying protocols were conducted in accordance with the WHO standard procedure and under constant supervision; operators wore WHO approved protective equipment when handling insecticide.

### Ghana

In Ghana this trial was conducted in Anwona (6°10’N, 1°43’06”W) and Mmemiriwa (6°14’N, 1°43’W), Obuasi Municipality. Obuasi Municipality is a gold-mining community in the Ashanti District, south-central Ghana. The climate is tropical with a mean annual temperature of 26°C and two rainy seasons extending from May to July and from September to November [18]. Malaria is hyper/holoendemic with intense perennial transmission by members of the *An. gambiae* complex and *Anopheles funestus*[19]. In 2005 approximately 11,000 malaria cases per month were recorded in Obuasi Municipality, affecting over 40% of AngloGold Ashanti gold mine employees, contractors and dependents. Consequently, in 2006 AngloGold Ashanti initiated an integrated malaria control programme, comprising vector control (IRS, LLINs and larviciding) and case management, supported by increased disease surveillance and health education. By 2007 reported cases had declined by 73% [20].

Blue DLs were installed in 28 randomly selected houses in Anwona and 32 in Mmemiriwa (Figure 2). To assess any differences between wall substrates, in both villages six houses were mud rendered and six houses were constructed from cement. In twelve additional houses in Anwona, all interior walls were sprayed with K-Othrine® WG 250 (25% w/w deltamethrin water dispersible granules) (Bayer Environmental Science) at an application dose of 25 mg/m^2^ for six mud rendered houses and 20 mg/m^2^ for six cement houses using a standard 15L Hudson X-pert pump.

### Mali

**Figure 3:**
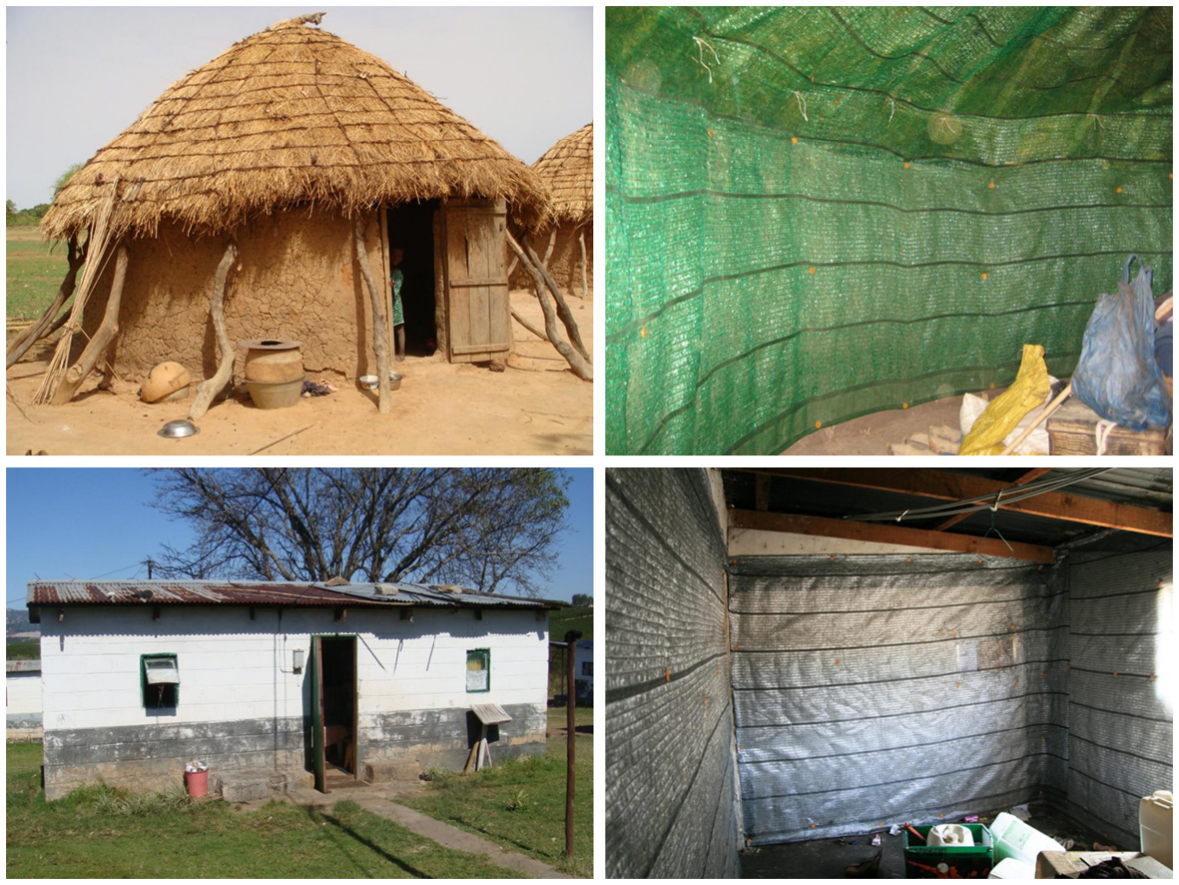
Durable linings (right) installed in traditional houses in rural Mali (top left) and South Africa (bottom left).

Mali in West Africa has a population of over 13 million inhabitants, of which 12.7 million are at risk of malaria [1]. The climate is subtropical in the south and arid in the north, with a hot and dry season from March to May, a rainy humid season from June to November, and a cool dry period from December to February. Malaria, principally transmitted by *An. gambiae s.s.* and to a lesser extent by *Anopheles arabiensis,* is endemic in the southern part of the country with seasonal peaks between May and November [21]. In 2006 an estimated 4.3 million cases were reported [22]. Green DLs were installed in 24 randomly selected houses in N’Galamadibi (13°33’N, 7°26’W), Banamba district of the Koulikoro region, 130 km northeast of the capital city Bamako. DLs were installed in a range of traditional Malian houses, including six square houses built from mud bricks with mud and wood roofs, eight round mud brick houses with thatched roofs, and ten square houses constructed from mud bricks, coated with mud and sand, with metal roofs (Figure 3).

### South Africa

This study was conducted on a private farm situated 10 km from Nelspruit Central Business District (CBD) of Mpumalanga, Eastern Transvaal Lowveld (25°27’23.83”S 31°00’12.17”E). Mpumalanga lies in eastern South Africa, north of KwaZulu-Natal, and borders Swaziland and Mozambique. Mpumalanga has a humid subtropical climate due to its proximity to the Indian Ocean, with an average annual temperature of 27°C. Malaria is endemic in these low-altitude areas with an estimated 4.3 million individuals at risk [23]. All study houses were rectangular and unplanned with a dining room/kitchen and two bedrooms. Houses were constructed from air bricks and cement with concrete floors, corrugated iron roofs and no open eaves. DLs were installed in single bedrooms in twelve houses. Five houses received silver DLs, four blue and three green (Figure 3). In four houses with DL installations, Fendona 6® SC (5.93% w/w alphacypermethrin) (BASF) was sprayed on all walls in a different room at an application dose of 20 mg/m^2^.

### Vietnam

**Figure 4:**
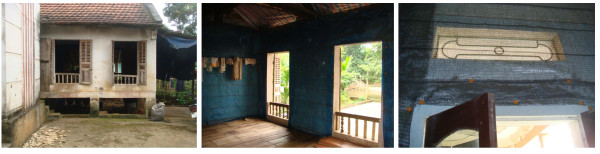
Durable lining installed in bedrooms (middle) or sitting rooms (right) in traditional houses in rural Vietnam (left).

DLs were installed in six houses in each of two rural villages located 65 km southwest of Hanoi in Hoa Binh Province, Kỳ Sơon district, Phuc Tien commune (20°56’23.92”N, 105°23’28.58”E), northern Vietnam. Hoa Binh Province consists of 10 districts, divided into 211 communes, with approximately 250,000 people considered at risk of malaria [24]. The climate is tropical to the south and monsoonal in the northern highlands, with a rainy season from May to September and a warm dry season from October to March. Despite concerted efforts by the National Malaria Control Programme (NMCP) in the mid 1990s, malaria transmission by *Anopheles sundaicus* continues in endemic coastal foci and by *Anopheles dirus* and *Anopheles minimus* throughout rural forested areas in the north [25]. Following observations of a number of construction differences between traditional Vietnamese sitting rooms and bedrooms, in each village DLs were installed in the bedrooms of three houses and in the sitting rooms of another three houses (Figure 4). Two houses in each village received blue DLs, two green and two silver. Six additional houses in each village were treated with K-Othrine® EW20 (2% w/w deltamethrin) (Bayer Environmental Science) at an application dose of 20 mg/m^2^ on all walls, and a further two houses in each village received untreated bed nets.

### User acceptability and durability

After installation, heads of households were interviewed at regular intervals throughout the year to monitor the levels of user acceptability and durability of DL under field conditions. Acceptability was assessed using a pre-tested, standardized questionnaire and verbal consent was obtained before beginning each interview. The questionnaire contained 38 questions pertaining to six general topics: installation, exposure reactions, entomology, indoor environment, aesthetics and durability. The majority of questions were closed and categorized, with examples including *“Have there been any changes in the number of mosquitoes in and around the house?”*, *“Did anyone from the house unit experience ill health after the installation?”* and *“Would you like to have the DL for decoration if it did not kill mosquitoes at all?”.* At the end of the interview participants were offered the opportunity to give open feedback about their wall treatments and general knowledge of malaria. At each follow-up interviewers surveyed the house to evaluate the condition of the wall installation and to record failed fixings, deformed yarns, material damage and evidence of lining cleaning.

Houses in Ghana (n=72) and South Africa (n=12) were interviewed at three weeks and nine months post-installation (Figure 1). In Mali (n=24) and Vietnam (n=28) follow-up occurred at one, five and ten months and one and nine months, respectively. The most frequent assessments were conducted in Equatorial Guinea (n=80) every three months throughout the year. The same questionnaire was used in all study sites at every time point. Respondents’ answers were grouped across time points and study sites for analysis, as indicated.

### Bioefficacy

Bioefficacy of vector control installations was evaluated using WHO cone bioassay tests according to the standard protocol [26] but with some variation in exposure time and interval between consecutive tests depending on the substrate. WHO bioassay cones were attached to DL-covered or IRS-sprayed walls *in situ* and ten mosquitoes were exposed for 30 minutes, with the exception of tests in Mali, where exposure time was 10 minutes. Three replicates were performed per household. In Equatorial Guinea, to evaluate the LLITCs, bioefficacy was measured using five mosquitoes per cone, five replicates per household, with three minutes exposure time. Mosquito mortality was recorded after 24 hours. All bioassays were conducted on the same wall surface in each test house at successive time points.

In Equatorial Guinea bioefficacy of DL, LLITCs and IRS (five houses per intervention) was measured at three, six and twelve months post-installation using laboratory susceptible *An. gambiae s.l.* (Figure 1). In Ghana bioefficacy of DL and IRS was assessed monthly using both field (Akaporiso, Brofoyedru or Amamom strains) and laboratory susceptible *An. gambiae s.s.*(Kisumu) in twelve houses, six representing each wall substrate (mud or cement); measurements of IRS efficacy ceased after six months.

In Mali cone tests were conducted in twelve DL houses at two, three, six and ten months following installation, using *An. gambiae s.s.*(Kisumu). In Vietnam bioefficacy of DL and IRS was recorded at one, three, six and fifteen months, using pyrethroid-susceptible *An. dirus* (Phu Khanh), in three houses from each intervention group (Figure 1). Bioassays performed at the South African site did not comply with the WHO recommended protocol and thus are not reported here.

### Chemical analysis

Determination of deltamethrin content in the field DLs was undertaken at the Vietnam study site by the Vestergaard Frandsen laboratories using High Performance Liquid Chromatography (HPLC) according to the Collaborative International Pesticides Analytical Council (CIPAC) protocol [27]. Before house installation and after one, three, nine and fifteen months, two 0.5 × 0.5 m samples of DL were cut from each house and total insecticide concentration per sample was measured in g/kg.

### Statistical analysis

Bioefficacy of DL and IRS in Equatorial Guinea and Ghana, as recorded using WHO cone bioassay tests, was analysed using a random effects logistic regression model, which took into account between-house variation (within-house correlation of bioassay results) at successive periods post-intervention. To compare across study sites, monthly bioassay measurements from Ghana were grouped into two categories: 1-3 months and 4-6 months after installation. The database of bioassay results was expanded to individual mosquitoes before applying the model. Odds ratios with 95% confidence intervals (CI) for bioassay mortality of the intervention (DL) relative to the control (IRS), adjusted for house, were estimated at three, six and twelve months after DL installation or spraying. Bioefficacy data from Mali were summarized comparing mosquito mortality proportions for DL over time because no IRS reference data were available. For Vietnam only mean mosquito mortality was recorded. All data analyses were conducted in STATA/IC 12.1 [28].

### Informed consent and ethical considerations

Volunteers from all study villages were recruited after obtaining written consent from community leaders/village committees and heads of households. All consent forms were reproduced and verbally explained in the local *lingua franca* to ensure volunteers understood the forms and all aspects of the study were described. Participants were informed that their involvement was completely voluntary and that they could withdraw from the trial at any time without penalty. In addition, at the time of recruitment, leaflets detailing symptoms of malaria and recommended precautions (including other forms of vector control) were distributed. Houses were each designated a number to maintain anonymity and all questionnaire answers were entered into databases using only this unique number as an identifier. This study received approval from the Ministries of Health in Ghana, Mali and Equatorial Guinea and the Ethics Committee of the London School of Hygiene and Tropical Medicine.

## Results

### Household installation characteristics

Across all five study sites a total of 220 households participated in this trial. DL was installed in 148 households, IRS in 48 households, LLITCs in 20 households and untreated mosquito nets in four households. By nine months post-installation six DL houses had withdrawn from the study. In Mali (n=24) three houses removed their DLs to renovate their rooms and two families were unavailable for follow-up. In South Africa (n=12) one farm worker had completed his contract and left, taking the DL with him. There were no reported drop-outs from Ghana, Equatorial Guinea or Vietnam. No household described a problem with the installation from any study site. The average surface area of DL and number of fixings used in each African study site are detailed in Table 1.

**Table 1:** Summary of installation features for African study sites

**Study site**	**No. of DL households**	**Average surface area of DL per room in m**^**2**^**(standard deviation)**	**Average no. nails used per meter of DL (standard deviation)**
**Equatorial Guinea**	40	42.8 (± 8.4)	1.5 (± 31.7)
**Ghana**	60	33.5 (± 2.0)	3.1 (± 14.4)
**Mali**	24	32.2 (± 2.1)	2.6 (± 24.5)
**South Africa**	12	12.6 (± 0.4)	7.4 (± 10.8)

The time taken to install DL was recorded in Mali and Vietnam. In Mali, where houses were single rooms, the average installation time for a three person team was 58.9 minutes (standard deviation of 19.4 minutes). In Vietnam, where DL was installed in either sitting rooms or bedrooms, the average installation times were 81.7 minutes and 77.5 minutes (standard deviations of 30 and 24 minutes), respectively. Vietnam was the only location where IRS application time was recorded; it took approximately 60 minutes for an individual to spray one house.

### *In situ* bioassays

**Figure 5:**
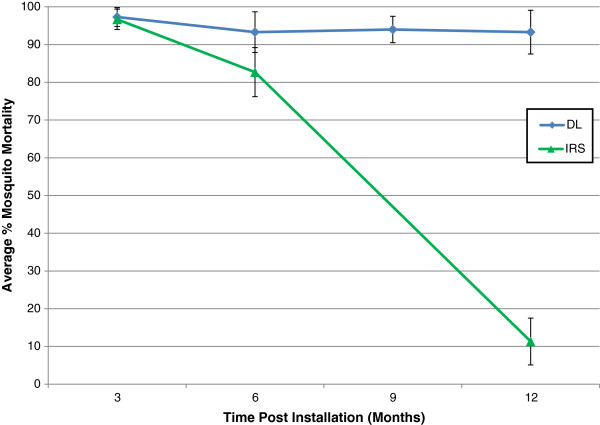
**WHO bioefficacy tests conducted with *An. gambiae s.l. *on DL and IRS interventions during the follow-up year in Equatorial Guinea.**

Across study sites and at all time points, DL demonstrated higher levels of efficacy than IRS, as measured by mosquito mortality (Table 2). In Equatorial Guinea a significant loss of IRS activity compared to DL was observed by six months post-installation (OR = 3.0, 95% CI 1.25 – 7.21, *p *= 0.014) (Figure 5). By contrast, no significant loss of DL bioefficacy was observed after twelve months of field use (OR = 0.93, 95% CI 0.83 – 1.03, *p *= 0.17). Mosquito mortality in both DL and LLITC households remained very high throughout the follow-up, with no significant difference in loss of efficacy between interventions after three (OR = 1.7, 95% CI 0.3 – 9.4, *p *= 0.55), six (OR = 2.7, 95% CI 0.5 – 13.8, *p *= 0.24) or twelve months (OR = 4.0, 95% CI 0.63 – 26.0 *p *= 0.14).

**Table 2:** Summary of bioefficacy results from Equatorial Guinea and Ghana

**Study site**	**Mosquito species**	**Wall substrate**	**Time post-intervention (Months)**	**Intervention**	**% Mosquito mortality (Total tested)**	Adjusted Odds Ratio^*^(95% CI) for DL mosquito mortality relative to IRS	***P *Value**
**Equatorial Guinea**	*An. gambiae s.l.*	Wood	3	IRS	96.7 (150)	1	
				DL	97.3 (150)	1.28 (0.31 – 5.18)	0.733
			6	IRS	82.7 (150)	1	
				DL	93.3 (150)	3.0 (1.25 – 7.21)	0.014
			12	IRS	11.3 (150)	1	
				DL	93.3 (150)	119.5 (47.2 – 302. 9)	<0.0001
**Ghana**	*An. gambiae s.s.* lab strain	Mud	3	IRS	98.2 (493)	1	
				DL	99.9 (998)	18.6 (2.23 – 155.4)	0.007
			6	IRS	69.4 (509)	1	
				DL	99.9 (966)	430.8 (59.6 – 3114.6)	<0.0001
		Concrete	3	IRS	99.8 (486)	1	
				DL	99.9 (1026)	2.11 (0.13 – 33.8)	0.60
			6	IRS	94.2 (536)	1	
				DL	98.7 (1017)	24.2 (2.30 – 254.8)	0.008
	*An. gambiae s.s.* field strain	Mud	3	IRS	90.6 (862)	1	
				DL	97.9 (1845)	4.93 (3.32 – 7.32)	<0.0001
			6	IRS	59.5 (975)	1	
				DL	93.3 (1916)	9.52 (7.60 – 11.92)	<0.0001
		Concrete	3	IRS	96.2 (867)	1	
				DL	96.7 (1809)	1.16 (0.76 – 1.79)	0.50
			6	IRS	85.9 (783)	1	
				DL	95.4 (1835)	3.38 (1.61 – 7.08)	0.001

^*^Values are adjusted for variation between house and bioassay replicate.

**Figure 6:**
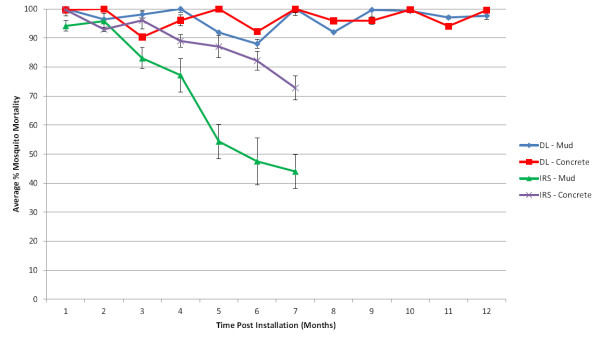
**Monthly WHO bioefficacy tests conducted with field *An. gambiae s.s. *on DL and IRS interventions during the follow-up year in Ghana.**

In Ghana the odds ratio of mosquito mortality for DL (relative to IRS) on mud walls for both lab (Kisumu) and field (Akaporiso, Brofoyedru or Amamom) strains increased as mosquito mortality for IRS declined over time with OR = 18.6 (95% CI 2.23 – 155.4, *p = *0.007) and OR = 4.93 (95% CI 3.32 – 7.32, *p *< 0.0001), respectively, at three months, and OR = 430.8 (95% CI 59.6 – 3114.6, *p *< 0.0001) and OR = 9.52 (95% CI 7.60 – 11.92, *p *< 0.0001), respectively, at six months (Table 2 and Figure 6). By six months, a significant increase in odds ratio of mosquito mortality for DL (relative to IRS) was also observed for lab and field strains exposed to DL on concrete walls with OR = 24.2 (95% CI 2.30 – 254.8, *p *= 0.008) and OR = 3.38 (95% CI 1.61 – 7.08, *p *= 0.001), respectively. No significant loss of DL bioefficacy was observed over the twelve month follow-up for either lab or field strain exposed to DL on mud with OR = 0.9 (95% CI 0.75 – 1.09, *p *= 0.27) and OR = 0.99 (95% CI 0.97 – 1.01, *p *= 0.33), respectively, or concrete walls with OR = 1.16 (95% CI 0.94 – 1.44, *p *= 0.17) and OR = 1.02 (95% CI 0.99 – 1.04, *p *= 0.25), respectively.

At the final follow-up in Mali and Vietnam (after ten and fifteen months, respectively), DL bioefficacy remained at 100%. However, in Vietnam, where IRS was applied to a subset of houses, average mosquito mortality fell to 60% after only one month and to 40% by three months post-intervention.

### Chemical analysis

Baseline DL field samples from Vietnam contained an average of 4.7 g/kg deltamethrin (range of 4.3-5.0 g/kg). After one month, the insecticide concentration had depreciated to 4.5 g/kg (range of 4.3-4.9 g/kg). By fifteen months in the field, samples of DL had retained on average 77% of their original deltamethrin content (3.6 g/kg, range of 3.4-3.8 g/kg).

### Adverse effects

No severe adverse effects were reported for any vector control product used during this trial. Over the twelve month follow-up a total of seventeen individuals from 214 households described a minor adverse reaction to an intervention. The majority of adverse reactions were reported for DL and the remainder for IRS (14/142 vs. 3/48, respectively, *p *= 0.45); none were reported for LLITCs in Equatorial Guinea. Most adverse responses (71%, 12/17) occurred within the first month after installation. Adverse effects, such as mild skin irritation (59%, 10/17), were the most common followed by serious skin irritation (6%, 1/17) and eye irritation (6%, 1/17). Forty-one per cent (7/17) of adverse reactions were reported for children under five years; the remainder were adults over fifty years (41%, 7/17) and adolescents (18%, 3/17). Of the effects reported for children under five, all infants were described as showing a special interest in the wall treatment and touching the wall more than previously. Adverse reactions were reported from households in Vietnam (7/28), Equatorial Guinea (6/80), Ghana (3/72) and South Africa (1/11). No side effects were reported in Mali.

### Household protection

A total of 100 households reported using other vector control products, of which ITNs (35%), repellent/coils (33%), untreated nets (21%), aerosols (6%) and electric traps (2%) were the most common choices. Use of personal protection products was described in Ghana (41/72), Equatorial Guinea (17/80), Mali (19/19) and Vietnam (23/28), but not from the South African study site. Coil/repellent use was not reported from Equatorial Guinea, which likely reflected the local product availability. By contrast, coil/repellents were the predominant form of alternative vector control used in Ghana (30/41 houses). Four per cent of households from all study sites described using more than one type of personal protection product, typically ITNs with repellents/coils. Three households also changed their choice of reported product during the twelve-month follow-up; two replaced repellents/coils with ITNs and one substituted ITNs for aerosols. Overall use of alternative vector control products was consistent across the twelve months (53% and 47%, before and after six months, respectively). In Equatorial Guinea, where more than two types of intervention were implemented, use of personal protection products was more prevalent in houses with LLITCs (7/20) than those with DL (8/40) or IRS (2/20).

### Householder perceptions of entomological effect

Throughout the twelve-month follow-up, DL households were interviewed at various intervals to assess any perceived changes in insect numbers and species composition. Overall 93% (313/336) of individuals interviewed had observed changes in the number of mosquitoes in and around their houses and 82% (250/304) reported experiencing fewer mosquito bites. Only 9% described a rise in number of bites. In addition, 85% (224/262) of respondents observed fewer species of nuisance insects inside their houses, in particular, cockroaches, termites, flies, moths, ants and spiders. All study sites reported consistent declines in mosquito numbers across all monitoring intervals (range of 88%, 114/130 in Equatorial Guinea to 98%, 117/120 in Ghana). However, only 18% (59/319) reported any dead mosquitoes in and around the DL. In Equatorial Guinea, where LLITCs were evaluated in a subset of households, 65% (52/80) reported changes in mosquito numbers, with 67% and 90% reporting fewer mosquito bites and other nuisance insects, respectively.

### Indoor environment

Eighty-three per cent (286/345) and 69% (238/347) of DL households from all study sites reported no change in indoor temperature or indoor light levels throughout the year, respectively. A total of 34 households reported an unusual smell post-DL installation, typically described as ‘the smell of insecticide’. Of these, only one house still experienced this smell one month after installation and only three houses expressed serious concerns with the smell. In Equatorial Guinea, 100% (78/78) and 96% (77/80) of LLITC households reported no change in indoor temperature or light levels throughout the year, respectively.

### Aesthetics acceptability

Three different coloured DLs were evaluated during this trial. All intervention houses in Equatorial Guinea and Ghana received blue DLs, households in Mali all received green DLs, and in South Africa and Vietnam blue, green and silver coloured DLs were distributed across the study communities. Individual households were allocated one DL colour throughout the trial. Overall, 97% (262/271) of individuals were satisfied with the colour of their wall lining. Considering each site individually, the DL colour each community received emerged as the most popular colour choice. For example, the majority of respondents in Equatorial Guinea (78/142) and Ghana (13/37) expressed a preference for blue DL, while the most popular colour choice in Mali was green (20/46). In Vietnam and South Africa the most popular DL colour was also blue (3/8 and 8/11, respectively).

When all sites were asked to identify the least desirable colour choice for DL, black and white emerged as equally unpopular (30%, 77/259 and 31%, 80/259, respectively), followed by red (19%, 48/259). Black was perceived to darken the house interior, white was considered too difficult to keep clean and red was disliked because of cultural connotations.

### Household acceptability

Sixty-six per cent (148/225) of participants agreed with the statement *“I would like to keep the DL for decoration even if it did not kill mosquitoes at all”* and 97% (326/335) wanted to keep their DL and have the installation repeated. Of those who wished to keep their DL, 73% (174/238) cited protective reasons and 20% (47/238) expressed a desire to keep theirs for decoration, while the remaining 7% (17/238) valued both qualities equally. Ninety-seven per cent (307/315) of individuals reported positive comments about their DL from house visitors. Many respondents also shared positive feedback when asked to describe their impressions of DL and knowledge of malaria (Table 3 details supporting quotations from Mali).

**Table 3:** Summary of malaria knowledge and perceptions of durable lining one month after installation in Mali

**Perceptions of DL**	**Knowledge of Malaria**
*“This fabric was very helpful because the mosquitoes have fled. The flies also leave us alone.”*	*“Malaria is a terrible disease and we pray hard that is disappears.”*
*“If this room has the textile, it will help protect those who sleep in this room from malaria.”*	*“…malaria is transmitted by the mosquitoes.”*
*“This installation is really good and I hope it can be done in other houses.”*	*“…malaria is a disease which is bad for both adults and children.”*
*“…textile has helped us a lot against mosquitoes and our hope is that they may be widely distributed throughout the village.”*	*“Malaria is a nasty disease that poses a huge problem in the rainy season and we worry especially for malaria in young children. Our hope is that it is eradicated.”*
*“The textile looks good in the room and protects against other insects, flies, cockroaches, etc.”*	*“Malaria is seasonal and not here for the moment. No infant has contracted malaria since you came.”*
*“You have done a great job and we will help you. I have sometimes suffered from malaria but now I do not suffer, nor do the children. I am at peace and rid of insects that kept me from sleeping well at night and resting in the afternoon. I am quite happy.”*	*“Malaria is transmitted by the mosquito bite. Oil also transmits the malaria.”*
*“I have nothing but blessings for you because I am comfortable in the house and even better without mosquitoes.”*	*“…malaria is a disease which causes vomiting.”*
*“Since the textile arrived I have not seen a case of malaria.”*	*“Malaria is a disease of poverty.”*
*“The textile truly reduces the density of mosquitoes in my rooms.”*	*“…malaria is a disease which changes the colour of your eyes.”*
*“The textile is very good because in addition to its insecticidal activity, it makes the room more beautiful.”*	*“Malaria is very bad because it kills.”*
*“The textile is a nice decoration.”*	*“Malaria is a serious disease and often deadly for children.”*
*“The textile kills the mosquitoes and makes my room beautiful.”*	*“I do not know of malaria.”*

All paired statements are reproduced from translated interviews with adult female house owners in N’Galamadibi, Mali.

In Equatorial Guinea, when offered the choice between DL, LLITCs or IRS, of all the respondents, 74% (161/218) chose DL, 22% (49/218) preferred LLITCs and 2% (5/218) wanted IRS. Regardless of which intervention was allocated initially, DL always emerged as the most popular vector control choice. Of those who received DL installations, 85% (106/125) favoured DL, 14% (17/125) LLITCs and 2% (2/125) IRS. Of those who received LLITCs, 56% (40/71) wanted DL, 37% (26/71) LLITCs and 3% (2/71) IRS. Of those who received IRS, 68% (15/22) preferred DL, 27% (6/22) LLITCs and 5% (1/22) IRS. Only three individuals (all from LLITCs houses) indicated that they wanted both insecticide-treated materials (DL and LLITCs).

### Physical durability

Over the course of the year there was evidence of some deterioration of the DL, the extent of which varied among countries. Wall lining damage occurred as a result of failed fixings and/or tearing of the material itself. Out of 142 DL households, 35 experienced failed nail fixings. Failures were only reported in Ghana (19/60), Mali (15/19) and Vietnam (1/12), with none reported from Equatorial Guinea (n=40) or South Africa (n=11). All houses in Mali (n=15) and Ghana (n=19) experienced failure of four nails or less. In Mali the majority of failed nails were observed one month after installation (7/15). Conversely, in Ghana the majority of failures were described at nine months post-installation (12/19).

Overall 23% (33/142) of houses experienced tearing of the DL (11/60 in Ghana, 11/19 in Mali, 10/40 in Equatorial Guinea and 1/11 in South Africa). No material damage was reported in Vietnam. In Mali and Ghana, 9/11 and 5/11 of the houses, respectively, which experienced nail failures also reported tearing of the wall lining. In Equatorial Guinea most tearing arose at twelve months post-installation (7/10), whereas in Mali the number of houses with damaged DL was equal at both one month and ten months (four houses at each). In Equatorial Guinea, where DL was installed using either nails and caps or bamboo poles, 8/10 houses which reported DL tearing had their installations fixed with nails.

Damage to the DLs predominantly arose at the doors, windows and room corners or at points of furniture contact, for example, bed frames. One house from Equatorial Guinea reported a heat lamp melting the DL and one house in Mali described a mouse eating the bottom of the textile. However, in the majority of cases, damage was attributed to the actions of children. The frequency of DL cleaning by householders varied between study sites. Overall 23% (32/142) of households reported cleaning their wall lining throughout the year. Cleaning was reported from Ghana (60/60), Mali (16/19) and South Africa (5/11) but not from Equatorial Guinea or Vietnam. Numbers of households cleaning their lining increased over time, rising by four-fold in Mali between one month and five months post-installation and by 57% in Ghana after nine months. Frequency of cleaning was predominantly weekly with either wet cloths or dry brushes. For households with LLITCs, 30% (6/20) reported cleaning their curtains by nine months.

## Discussion

In order to establish ZeroVector® DL as a viable long-lasting alternative for IRS, it must demonstrate equivalent or superior levels of bioefficacy, acceptability, durability and logistical feasibility to currently available products. In this study, superior efficacy was achieved by DL compared to IRS throughout the trial. In both Mali and Vietnam, complete mosquito mortality using DL was observed at ten and fifteen months post-installation, respectively. By contrast, loss of IRS activity was reported after only one month in Vietnam and by six months in Equatorial Guinea and Ghana. These results are explained by ZeroVector® DL containing a long-lasting insecticide reservoir, while IRS has finite residual activity. However, this trial was only conducted for one year and further evaluations must be undertaken to determine how efficacious DL remains after three to four years of field use.

The DL was received with high levels of acceptability, from both study participants and external visitors, as evidenced by numerous positive responses to entomological and aesthetic questions. Although the impact of DL on local vector populations was not empirically measured, perceived reductions in mosquito density, mosquito bites and nuisance insects were described from all study sites and across all time points. In addition, when asked if they would like to retain their DL even if it had no effect against insect vectors, the majority of participants agreed with this statement. This result suggests DL may be accepted by users based on its aesthetics alone. The importance of both perceived entomological efficacy and decorative value, as key determinants of household acceptability, has been previously demonstrated during early field evaluations of prototype wall linings [29]. Most participants in this study also did not express objections to the effect of DL on their indoor environment, reporting no changes in indoor temperature or light levels. An overwhelming majority of respondents wanted to keep their DL at the end of the trial and were satisfied with their lining colour. When offered a choice of vector control product in Equatorial Guinea, all intervention groups preferred DL to LLITCs and IRS.

Regarding durability, after one year, a minority of households reported failed nail fixings and/or damage to the DL, with one severe case described (burning of DL in Equatorial Guinea). The latter was unexpected as ZeroVector® DL has been subjected to flammability tests conducted by an independent agency (The Govmark Organization Inc.) and is considered non-flammable (documentation available from manufacturer on request). No household reported actively repairing failed fixings or lining tears, which may indicate that either the DL was not genuinely valued or passive recipients were not focused on making routine repairs at this stage of the intervention. The method and quality of DL cleaning varied widely between households and study sites; in Ghana all respondents reported cleaning their wall lining, while no such activity was described from Equatorial Guinea or Vietnam. In future, the installation of DL should be complemented with information about care and maintenance.

Currently, ZeroVector® DL is manufactured in large rolls (2.3 × 100 m) that require an external installation team to correctly cut for particular house dimensions and fix with nails to avoid wastage and ensure optimal physical durability. In Mali and Vietnam it took three individuals approximately 60-75 minutes to install DL in one house. This is comparable to the time taken by a single person to apply IRS to one house (~60 minutes) but with the advantage that DL may only need to be repeated every three to four years, not every six to twelve months. Additionally, in Equatorial Guinea, where DL was installed using bamboo rods sourced from local materials, this subset of houses reported fewer failed fixings and lining damage than those which received DL installed with nails. These observations suggest that with such high levels of acceptability and adequate supervision during installation, DL has the potential to be widely implemented at the community-level when supported by local technical and logistical infrastructure. Future improvements could include the production of DL rolls for specific house dimensions that are consistent among individual countries, cultures or communities.

The high DL efficacies reported in this study were supported by a loss of only 23% of deltamethrin content after fifteen months, a similar rate to recent field evaluations of LLINs. After twelve months of field use, a loss of 30% of alphacypermethrin from Interceptor® LLINs was reported [30], and 17% and 11% of deltamethrin from PermaNet® 2.0 and PermaNet® 3.0, respectively [31]. In addition, the DL demonstrated equivalent levels of efficacy in the WHO bioassays to the LLITCs in Equatorial Guinea, suggesting it remains as effective as other long-lasting netting products for at least one year in the field. However, unlike DL, the high LLITC bioefficacies did not result in as great a perceived reduction in mosquitoes. More respondents with DL described declines in mosquito numbers and bites than users of LLITCs, and more LLITC households reported using alternate vector control products which could be interpreted as dissatisfaction with their intervention.

While this present study does not seek to establish the mode of action of DL on vector populations, growing evidence suggests that DL is more comparable to IRS than LLINs. Experimental hut trials in Burkina Faso indicated mosquito mortality was the principal activity of permethrin-impregnated DL, and only slight inhibition of *An. gambiae* entry and feeding was observed [32]. Similar reports from Benin where bendiocarb-treated wall coverings also did not impact on blood feeding [33] suggest that wall linings afford little to no personal protection when used by only a minority of houses [34]. Instead, it is anticipated that DL will control malaria transmission when applied widely at the community-level, through its effect on mosquito density and longevity. In support of this, recent studies from India achieved a mass population effect on malaria vectors using high coverage of deltamethrin ITPS in temporary urban labour settlements [35] and tribal villages [14].

### Study limitations

There are several weaknesses in the reported study design, which need to be considered when interpreting the data. The questionnaires were designed to ask direct questions with standardized delivery to ensure consistency of responses between countries. It could be argued that some questions were too specific, or not sufficiently open, so that interviewees delivered the answers they anticipated the questioner wanted. In all study sites the interviewers were associated with the installation process, making it probable that a proportion of respondents’ answers were not as objective as they might have been had the interviewers been entirely unknown. Entomological indices, including indoor and outdoor resting catches of mosquitoes and blood meal ELISAs, are needed to corroborate the reported entomological effects of DL. This study lacked a control (i.e., a DL with no insecticide) and it is possible that even users of untreated DL would have perceived a decline in mosquito biting if that is what they expected to see. Wall installations may also have coincided with seasonal changes in mosquito biting and as such results are not adjusted for respondents’ preconceptions. Nevertheless, all study sites reported fewer mosquito numbers and bites at all time points across the year, suggesting that this was an observable phenomenon.

While IRS and LLITCs acted as control interventions, to a certain extent this trial would have benefitted from an untreated control had such a material been available. An alternative approach would have been to offer both treated DL and untreated DL as ‘aesthetic interventions’ with no mention of their control capabilities and then probe for entomological observations or perceptions during follow-up interviews. This study design would also allow the fourteen adverse reactions to DL to be attributed to this product and not to a ‘placebo effect’. However, there was reluctance to use an untreated control by authorities in some countries at a stage when the product was completely unknown. Finally, WHO cone bioassays are designed to measure the residual activity of an insecticide-treated substrate and not its efficacy against free flying vectors. Comparisons of indoor and outdoor mosquito densities, human landing catches or light trap collections, between intervention and control clusters in a community randomized trial, would be required to substantiate these basic efficacy results.

## Conclusions and recommendations

This is the first study to evaluate the efficacy, acceptability and durability of long-lasting insecticide-treated durable wall lining compared to conventional IRS in multiple field situations. After twelve months of field use, ZeroVector® DL remained fully efficacious against mosquito vectors and showed minimal loss of insecticide content. DL demonstrated superior levels of efficacy to IRS from six months onwards at all study sites. Less than one third of households experienced problems with failed fixings or lining tearing throughout the year. Importantly, DL was unequivocally more popular than IRS and other long-lasting vector control products trialed (LLITCs). Finally, this study demonstrates that DL has the potential to be widely accepted and executed across disparate environments, cultures and operational conditions, thereby supporting its application amongst a range of malaria endemic areas. Future studies are required to evaluate the community impact of DL compared to IRS and untreated DL in cluster randomized trials measuring vector population density, longevity, entomological inoculation rate and sporozoite rate, in conjunction with malariometric and disease control outcomes.

## Competing interests

The authors received financial support from The Mentor Initiative and Durable Activated Residual Textiles S.A. (DART S.A.) to conduct the study but have no competing or commercial interests with either company. Neither of these commercial parties played any role in data analysis, interpretation of results, decision to publish or preparation of the final manuscript.

## Authors’ contributions

The field trials were initiated by DART S.A. and The Mentor Initiative and conducted by AM, ANM, JBSO, SK, DAB, MBC, MLL, AST, BD, MK, AG, SFT, CEGM and HL. Data was consolidated, interpreted and analysed retrospectively and independently by LAM, IK and MR. LAM, IK and MR wrote the manuscript. All authors read and approved the final manuscript.
